# Public Health Genetics: Surveying Preparedness for the Next Generation of Public Health Professionals

**DOI:** 10.3390/genes14020317

**Published:** 2023-01-26

**Authors:** Anastasia M. Jacko, Andrea L. Durst, Karen L. Niemchick, Stephen M. Modell, Amy H. Ponte

**Affiliations:** 1 Department of Human Genetics, School of Public Health, University of Pittsburgh, Pittsburgh, PA 15213, USA; 2 Grand Valley State University, Allendale, MI 49401, USA; 3 Department of Epidemiology, Center for Public Health and Community Genomics, School of Public Health, University of Michigan, Ann Arbor, MI 48109, USA; 4 Genedu Health Solutions, Beaufort, SC 29902, USA

**Keywords:** public health, genetics, genomics, literacy, education, survey

## Abstract

Since the Human Genome Project’s completion in 2003, the need for increased population genetic literacy has grown exponentially. To address this need, public health professionals must be educated appropriately to serve the public best. This study examines the current state of public health genetics education within existing master of public health (MPH) programs. A total of 171 MPH Council on Education for Public Health Accreditation (CEPH)-accredited programs across the nation were identified via a preliminary internet search. The American Public Health Association (APHA) Genomics Forum Policy Committee created 14 survey questions to assess the current status of incorporating genetics/genomics education within MPH programs. Using the Qualtrics survey system through the University of Pittsburgh, a link to the anonymous survey was sent to each director’s email address obtained from their program’s website. There were 41 survey responses, with 37 finished to completion, for a response rate of 21.6% (37/171). A total of 75.7% (28/37) of respondents reported having courses containing genetics/genomics information in their programs’ coursework. Only 12.6% reported such coursework to be required for program completion. Commonly listed barriers to incorporating genetics/genomics include limited faculty knowledge and lack of space in existing courses and programs. Survey results revealed the incongruous and limited incorporation of genetics/genomics within the context of graduate-level public health education. While most recorded programs report offering public health genetics coursework, the extent and requirement of such instruction are not considered necessary for program completion, thereby potentially limiting the genetic literacy of the current pool of public health professionals.

## 1. Introduction

### 1.1. Rise of Public Health Genetics

While the importance of public health has been recognized since the John Snow era, the question of how genetics fits into that framework has only recently become a topic of concern. Since its inception in 1914, public health education has become essential to the generation of well-informed public health professionals, and such professionals are needed to protect the health and safety of their “communities through education, policymaking, and research for disease and injury prevention” [1]. As disease and injury are just as much part of genetic conditions as they are communicable conditions, accidents, and trauma [2], the role of genetics in public health has, in the last several decades, come under increasing attention.

With the advent of the Human Genome Project (1990–2003), scientists embarked on a comprehensive effort to sequence and understand the human genome. Concurrent with the initiation of the Human Genome Project, the Centers for Disease Control and Prevention (CDC) created the Office of Public Health Genomics (now the Office of Genomics and Precision Public Health) in an effort to integrate genomics into public health programs, policies, and research [3,4]. Omenn describes this rapidly developing field as follows: “Public health genetics is an exciting interdisciplinary area that brings all the public health sciences to bear on the emerging challenge of interpreting the medical and public health significance of genetic variation within populations” [5]. Clearly, the age of genetic medicine and precision health is upon us, and, as such, a properly educated public health workforce is required to educate, advocate, and protect the health of the public. Policies protecting individuals from genetic discrimination, minimizing the potential harm of genetic test results to the public while maximizing their benefits, guaranteeing the accessibility of appropriate genetic services, and offering population education in genetics, to name a few, have become items that professionals need to address. Therefore, a close examination of the current Masters of Public Health (MPH) programs in the United States for genomics content could shed light on whether public health professionals are obtaining the necessary knowledge to best prepare them for navigating this new era. 

### 1.2. Integration of Public Health Genetics into Practice

In 2000, a team of public health leaders came together to develop genomic competencies to address the increasing need for public health professionals to incorporate genomics into their practice. Areas of focus included applying the basic public health sciences to genomic issues, studies, and testing; learning the uses of genomics as a tool for achieving public health goals within one’s field of practice; the ability to participate in program evaluation and policy planning; and the ability to identify genetic testing ethical and medical limitations. These competencies were not created to replace the existing public health competencies but, rather, to enhance and update them in response to the rapid advancements in human genetics technologies. The ultimate goal of this project was to assure that public health practitioners would be able to take advantage of genomic advances and steer public health programs towards augmenting their curricula by incorporating genetics/genomics for the training of future public health leaders [6].

As early as 2002, the Institute of Medicine (now the National Academy of Medicine) acknowledged the existent genetics educational gaps as well as the growing need for the public health workforce to have sufficient knowledge of genetics and genomics, declaring that health professionals require these competencies to effectively address the future challenges facing society [7]. Further, the European Public Health Association has included among its training and education objectives “capacity building to develop a skilled, diverse, and dynamic public health genomics workforce and network of multiplicators” [8]. Likewise, the Working Group on Innovation and Good Practice in Public Health Education of the Association of Schools of Public Health in the European Region (ASPHER) has included public health genomics in the European Core Competencies for Public Health Professionals [9]. 

### 1.3. Public Health Program Core Competencies and Accreditation

In 2004, in conjunction with the CDC, the Association of Schools of Public Health (ASPH) initiated the Core Competency Model Development Project for the Master of Public Health (MPH) degree [10]. At the time, and still to this day, MPH programs have been focused around five core disciplines: health policy and management, social and behavioral sciences, epidemiology, biostatistics, and environmental health sciences. 

Initial ASPH efforts had an overall goal centered on standardizing discipline-specific competencies with one working group per core discipline, and the sixth focused on public health biology. The public health biology group was organized to address the gaps between the different educational backgrounds of those seeking an MPH since not all MPH candidates have a strong scientific base. Further work defined a set of cross-cutting domains, including public health biology [11]. However, future program accreditation guidelines aligning with this set of competencies exclude public health biology as it was defined, thereby missing genetics and genomics.

The American Public Health Association (APHA) was the first entity to formulate guidelines for accrediting schools of public health in the 1940s and remained the sole accreditor until 1973 when it transferred responsibility for MPH programs to the independent Council on Education for Public Health (CEPH) [12]. The ASPH Core Competencies served as the basis for the development of the CEPH MPH competencies: a subset of the CEPH Accreditation Criteria. The Criteria are broadly progressive in the sense that they include both genetic and environmental factors under foundational public health knowledge. However, it is noted in the CEPH Accreditation Criteria that these topic areas included under foundational public health knowledge “are defined in a more granular, less advanced level than the competencies typically used to define outcomes of a graduate-level program of study” and at no point in their description of MPH competencies does the Accreditation Criteria cite genetics/genomics, which can influence those competencies expected of MPH students by schools of public health in the United States [13,14]. This shortcoming could result in the perception that the topic of genetics/genomics is expendable, downplaying the importance of genetic literacy for working public health professionals. 

### 1.4. Public Health Genetics Programs

In Europe, genetic epidemiology has become a part of public health curricula at several institutions, including the Erasmus University Medical Center in the Netherlands and the Institute of Hygiene and Preventative Medicine at the Universita Cattolica del Sacro Cuore in Italy [15]. Socio-ethical genomics coursework has been incorporated into curricula at Erasmus University as well as the University of Deusto and the University of the Basque Country, Spain. As the overlap between genetics and public health in practice continues to grow, only three schools of public health in the United States have implemented dedicated programs in public health genetics. These programs could provide a useful precedent. Organized curricula exist at the University of Michigan: Public Health Genetics Interdepartmental Concentration (PHGIC) [16], the University of Washington: Master of Public Health Genetics [17], and the University of Pittsburgh: Master of Public Health Genetics [18]. Each program participated in the multi-institutional Genetics in Public Health Training Collaboration, which was pivotal in developing the scope of public health genetics education and training [19]. Although these programs each have concentration-specific competencies in public health genomics, CEPH’s MPH Concentration Competencies called for programs to define at least five distinct competencies for each concentration and still leave little room for the inclusion of genetics/genomics in other program concentration competencies considering the widespread focus on ASPH’s five ubiquitous core disciplines [13,16].

Because of the history and ongoing need for public health professionals to be literate in public health genomics, we conducted a survey to explore the current state of genetics/genomics education within the existing CEPH-accredited MPH programs across the United States. This paper covers details of the survey, findings with respect to the inclusion of public health genomics into existing curricula, how well courses prepare public health students in genomics competencies, and educational strategies that might be adopted in the future.

## 2. Materials and Methods

### 2.1. Data Collection

Our survey was developed in coordination with the APHA Genomics Forum Policy Committee. The population of interest was identified through an Internet search of the current CEPH U.S.-accredited MPH programs. As of 7 May 2019, 171 accredited programs were listed on the CEPH website (https://ceph.org/about/org-info/who-we-accredit/accredited/#programs (accessed on 7 May 2019)). Email addresses for current program directors were found by going to each program’s website.

### 2.2. Survey Design

Survey questions were developed to ascertain whether public health genetics was part of the existing program curriculum, the titles of relevant courses, whether the courses fulfilled CDC competencies and barriers to implementing additional public health genetics content in the future. The survey was reviewed by the University of Pittsburgh IRB and was found not to require IRB oversight. A series of 14 questions were included in the survey. The questions fit various types: true/false, multiple choice, open-ended, and slide bar, and included skip logic. The question about barriers to implementation was hybrid, containing both fixed choices and opportunities to elaborate on other possible obstacles. The questions were entered into Qualtrics [20], and anonymous links were conveyed via email to the directors of the 171 CEPH-accredited programs. The initial email was sent out on 17 September 2019, and two subsequent reminder emails were sent on 25 November 2019 and 1 December 2019.

### 2.3. Data Analysis

Descriptive statistics were generated for the survey data. Geographic regions (Midwest, Northeast, Northwest, Southeast, Southwest), where the respondent’s institution was located, and its urbanicity/rurality was tabulated. MPH program factors were also examined, including the number of years the MPH program had been in existence and whether it was a medical school or hospital affiliation. The characteristics of the MPH program curricula of the survey respondents included whether the program offered one or more of the following: A. introductory courses in public health genetics/genomics; B. courses focused on specific areas in genetics/genomics; and C. courses that incorporate some topics (as part of a course) in genetics/genomics to MPH students. Summary statistics were determined along a 10-point Likert scale indicating the degree to which the curriculum prepares students to address the CDC Genomics Competencies. 

The Fisher exact test of independence was performed between a lack of faculty knowledge of genetics/genomics and three types of barriers to the incorporation of genetics/genomics into the curriculum. This test was used based on sample size. All analyses were performed in STATA v. 16 [21].

## 3. Results

### 3.1. Respondent Characteristics

In total, 41 program directors responded to the questionnaire, of which four were deemed unfinished by Qualtrics and considered unusable, for a response rate of 21.6% (37/171). Of the 37 qualifying respondents, 25 program directors reported the geographic region where their institution was located, with the majority being in the Northeastern United States (28%). The others were less prevalent: Midwest (24%), Southeast and Southwest (both 20%), and Northwest (8%). All 37 respondents noted the years that their MPH programs have been in existence, with the majority having existed over 11 years (81.08%), followed by 6–10 years (16.22%), and lastly, 1–5 years (2.70%). Other demographic characteristics of the 37 respondents are summarized in Table 1. 

### 3.2. Genomics Competencies

For questions related to the CDC Genomics Competencies, not all individuals responded to a particular competency. Of the 37 survey respondents considered in our analysis, the number recording an answer for a given competency ranged from 20 to 25 individuals (see Table 2). Survey respondents marked along a scale of 1 to 10 for each competency, with 10 being the most prepared, to answer how well the MPH curriculum prepared their students to address the eight CDC Genomics Competencies. All response means were less than six. The lowest mean scores were for competency C—staying up to date on genetic advances relevant to their specialty and incorporating that knowledge as a tool to achieve public health goals related to their area of practice (3.36), and for competency E—participating in strategic policy planning and development related to genetic testing or genomic programs (3.35). Table 2 contains a summary of the data related to our CDC Genomics Competencies findings.

### 3.3. Course Offerings

All 37 respondents answered questions relating to the characteristics of their MPH curricula and course offerings. One or more introductory courses in public health genetics/genomics were reported as available to MPH students in 37.84% of respondents’ institutions. Of those respondents who noted that these courses were available, 71.43% (n = 10) reported that these courses were not required, 14.29% (n = 2) said that all students were required to take these courses, and 14.29% (n = 2) reported that these courses were required for some students. One or more courses focused on specific areas in genetics/genomics were reported as being available to MPH students in 51.35% (n = 19) of respondents’ institutions. Of those respondents noting that such courses were available, 73.68% (n = 14) reported that these courses were not required, 5.26% (n = 1) reported that these courses were required for all students, and 21.05% (n = 4) reported that these courses were required for some students. Finally, one or more courses that incorporated some topics (as part of a course) in genetics/genomics were reported as being available to MPH students in 70.27% of respondents’ institutions and unavailable in 29.73% of respondents’ institutions. Of those respondents noting that such courses were available, 38.46% reported that these courses were not required, 42.31% reported that these courses were required for all students, and 19.23% reported that these courses were required for some students. 

### 3.4. Curricular Challenges

**Figure 1:**
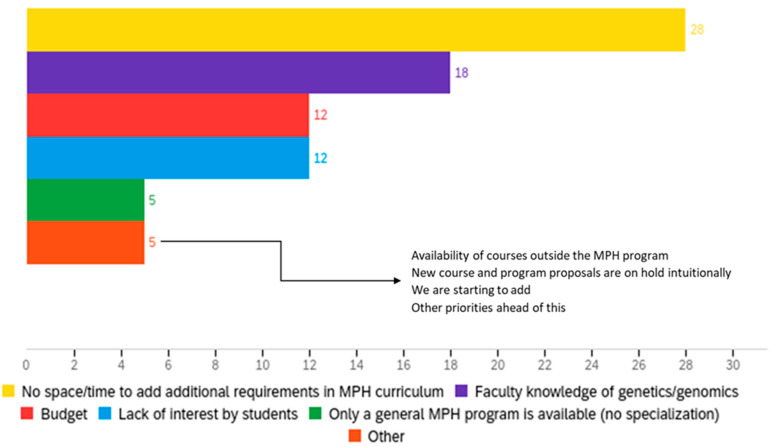
Reported barriers to incorporation of genetics/genomics in existing MPH programs.

Thirty-six participants reported barriers to the incorporation of genetics/genomics in their existing programs; one participant refrained from answering this question. The barrier selected most often was a lack of time and space to include additional requirements in their MPH curriculum (77.78%, n = 28), followed by 50% (n = 18) reporting a lack of faculty knowledge in genetics/genomics. Fifteen programs (41.67%) selected one or more barriers to the provision of genetics/genomics content in their program. Other barriers that were identified included limited knowledge of the existing faculty, budget, and lack of student interest. Five programs identified barriers beyond those provided on the survey, including courses that existed outside the MPH program for students to take and how priorities other than genetics/genomics took precedence in the curriculum (Figure 1).

As shown in Table 3, the results indicated a statistically significant increase in the proportions of respondents including: (1) one or more introductory courses in public health genetics/genomics for those who reported not being challenged by faculty knowledge of genetics/genomics [prevalence of 63.2% (12/19)], compared to 11.1% (2/18) who reported being challenged by it (*p* = 0.002); (2) one or more courses primarily focused on specific areas in public health genetics/genomics in those who reported not being challenged by faculty knowledge of genetics/genomics [prevalence of 73.7% (14/19)], compared to 27.8% (5/18) who reported being challenged by it (*p* = 0.009); and (3) one or more courses with some topics in public health genetics/genomics for those who reported not being challenged by faculty knowledge of genetics/genomics [prevalence of 89.5% (17/19)], compared to 50% (9/18) who reported being challenged by it (*p* = 0.013). 

For those who stated that their programs incorporated genetics/genomics in their MPH courses, titles spanned all five domains, with the majority being in epidemiology. The titles included: Genetic and Molecular Epidemiology; Ethical, Legal, and Social Issues in Genomics and Health; Genetics, Health Behavior and Health Education; High Throughput Molecular Genetic and Epigenetic Data Analysis; Nutrigenomics; and Biological Genetics in Public Health. All reported course titles are listed by category in Table 4.

### 3.5. Open-Ended Responses

The final question of the survey simply asked: “Do you have any other questions or concerns regarding this topic or this survey?” The majority of those that answered the final open-ended question reported the fact that the mid-south was missing from the regional location question. One respondent wrote, “Will the results of this survey be made broadly available? I would like to see the results.” Another comment, especially, stood out: “This is an important subject but given the current emphasis of most public health programs and accrediting agency, one that is unlikely to be developed soon.”

## 4. Discussion

### 4.1. Possible Strategies

The final, open-ended comment underscores the importance of the study. When CEPH compiled the list of requirements for MPH program accreditation in 2011 (and again in 2016 when the competencies were reassessed), the master’s competencies did not include genetics/genomics [22]. Given this absence, it is not surprising that this survey revealed that the majority (62.16%, n = 23) of responding CEPH-accredited programs do not have an introductory course specific to public health genetics. Additionally, even though the majority of the programs stated that they had courses containing some sort of public health genetics element (75.7%, n = 28), their perceived confidence level in how well these programs prepare their students on the CDC genetics/genomics competencies was low, made evident by a maximum mean indication of preparedness for any of the competencies at 5.88 out of 10 (Table 2). These results align with Marzuillo et al. in their survey of Italian public health professionals, which revealed that their perceived genetics/genomics knowledge base required improvement [23]. This relatively low level of perceived confidence in student preparation related to public health genetics/genomics among programs illustrates the importance of aligning the CDC and CEPH guidelines for program accreditation. A change in the CEPH guidelines would likely precipitate curriculum change in MPH programs. If the curriculum related to public health genetics were to increase in MPH programs, one could anticipate that such a shift would promote an increase in the level of confidence among program directors in their ability to provide the necessary comprehensive public health genetics instruction to their students. 

The incorporation of genetics/genomics material into MPH programs can be accomplished via a multitude of methods, tailoring integration in whatever fashion best fits an existing program. Guidance can be found in the first two public health genetics programs at the University of Washington and the University of Michigan; the former developed a sixth discipline with the main focus being genetics/genomics, and the latter incorporated genetics/genomics in a multidisciplinary manner [16,17]. Genetics/genomics can also be introduced into the curriculum through existing courses or through upper-level courses that focus on one aspect of public health genetics. These developments are already taking place at a number of institutions, as exemplified by the courses with genetics/genomics components found in Table 4. 

Each of the five disciplines of public health had at least one genetics/genomics-related course title associated. However, it is important to take note of study findings, such as those of Ianuale et al., when designing these courses, as the material must be pertinent to the evolving field of genetics/genomics and not just cover historical/classical genetics [9]. The most practical solution for incorporating public health genetics into MPH programs would be to intersperse genetics/genomics into existing courses, tailoring the information in a discipline-specific manner, or to create a course specific to public health genetics that is part of the required course load for all MPH students, since the most important goal is to expose and prepare all future public health professionals to public health genetics regardless of specialty. 

This survey revealed that limited time and space allotments within existing programs were the largest self-reported barrier to the incorporation of genetics/genomics into public health curricula. The second largest barrier to genetics/genomics incorporation was a lack of faculty knowledge in the field, which aligns with recent studies on the attitudes and genetic/genomics knowledge base of public health educators, as explored by Chen et al. [24,25]. To combat this barrier, the development of higher education programs and continuing education programs that cover the topic of genetics/genomics is paramount. By educating the educators, public health students and educators can become more genomically literate public health professionals. Collaboration has value—guest lecturers could be brought in for seminars or a number of classes to expose students to the importance of genetics/genomics without putting pressure on the existing faculty.

### 4.2. Public Health Implications

Knowledge of the role of genetics and genomics in a number of chronic diseases continues to grow, illustrating the importance of public health professionals having familiarity with genetic and genomic concepts. A survey of state chronic disease directors showed that 11/16 considered program integration of CDC Tier 1 genomic applications, such as the use of family history and testing for hereditary breast and ovarian cancer (*BRCA1/2* genetic counseling and testing) and Lynch syndrome, to be beneficial to individuals and families in their state [26]. Through their involvement in public health education, the authors are knowledgeable of several programs that have implemented public health coursework that considers these two conditions from the perspective of the genetics involved, lifetime risks, and family tracing, i.e., cascade screening. Students wrestle with risk management and program implementation scenarios that prepare them for professional decision-making. Applied knowledge of heritable conditions gives public health professionals the acumen to participate in state programs, such as those previously funded by the CDC to support genomics screening and public/professional education in seven states [27], and to raise public awareness of heritable conditions and services during their own practice. With their focus on health equity, public health professionals have an important role in increasing access to health services, including genetic services.

Additionally, genetics and genomics represent growing areas within several public health specialties [28]. Omenn pointed out that the field of public health genetics is expanding beyond traditional clinical conditions to include those that impact environmental health, nutrition, infectious disease (HIV and COVID-19 being the latest examples), and unhealthy behaviors, such as tobacco and other substance use [29,30]. These practical areas, considered in the genetic context, should provide support for the enhancement of what is currently taught in schools of public health. 

For example, the COVID-19 pandemic offers a unique opportunity to bring together genetics and immunizations: one of public health’s “top 10 public health achievements of the first decade of the 21st century” [31]. The unanticipated and sudden chain of events from the start of the pandemic to the rolling out of mRNA vaccines thrust public health into the forefront and necessitated public health professionals to be knowledgeable in basic cellular genetics as well as basic immunology to educate and inform the public. Health messaging seems to have moved beyond the immunization campaigns of the past into a more complicated landscape to maneuver through. The new set includes complex biomedical technologies coupled with more messaging avenues than ever before. The MPH curricula that include such material are becoming imperative as oncoming national and international health challenges necessitate both medicine and public health to incorporate genomics into their respective practices [32].

Genomic literacy on the part of public health professionals translates into the ability to help healthcare professionals and the public more effectively. An APHA policy statement on genomic literacy outlines this information-sharing role, which includes helping to train healthcare personnel and community health workers in their awareness of genetics and genetic services (e.g., of genetic risk and use of family history), providing information and sponsoring lectures for health professionals and lay communities, and providing genetic and genomic information and resources to hospitals, physicians’ offices, and laboratories [33,34,35].

In addition to the training the trainer effect of public health genetics education that we emphasize, it also enables public health professionals to more effectively fulfill public health’s core functions—assessment, assurance, and policy [36,37]. CDC’s Genomics Competencies for the Public Health Workforce reflect these areas, e.g., evaluating the knowledge of prevention measures, planning for genomics services, ensuring laboratory testing standards, the ability to explain risk and benefit in health and disease assessment, and collection of genetics-related data [6]. Genetics and genomics education is a key ingredient in enabling these skills to be integrated into the current activities of the public health workforce.

### 4.3. Limitations

Although our study revealed some interesting findings regarding the current state of genetics/genomics education in MPH programs, limitations exist in its response rate, scope, and reach. Only 37 out of 171 directors completed the survey, which, therefore, may not provide a full picture of the status of the incorporation of public health genetics into MPH programs.

Authors have noted the impact of the response rate on the possibility of response bias, which can skew the accuracy of survey findings [38,39]. The authors took steps to preemptively address three kinds of factors that could influence the response rate in the survey methodology employed: (1) design stage issues, such as question item language, question/response format, and survey length; (2) misidentification of the target person receiving the survey; and (3) lack of re-contact. We were able to partially address these concerns through multiple rounds of grammatical and question item adjustment between APHA Policy Committee members and updating the MPH program directors list from the CEPH website before emails were sent out. However, the anonymization of recipients in Qualtrics restricted our ability to make follow-up inquiries with nonrespondents (the second survey was e-mailed to all recipients, respondents and nonrespondents alike). Modification of the stage at which anonymization takes place will be necessary for future surveys to allow the assessment of the exact reasons for nonresponse.

Chen et al. conducted a large online survey of public health genomics knowledge in public health educators within the United States [40]. Their use of four organizational membership listings, an initial survey notice, and two follow-up messages succeeded in boosting the number of respondents to 1862 public health educators. However, their adjusted response rate, taking into account inclusion criteria, was 23.1%: similar to our 21.6% response rate. The comparability of the two figures suggests that online surveys embodying a novel theme, such as genetic applications, and targeting professionals operating within a highly defined scope of practice and training may need to think outside the box to heighten the response rate, e.g., by organizing focus groups of professionals to inform the survey, use of organizational leadership and snowball techniques to aid sample recruitment, and a consideration of the time of year when distributing surveys to busy professionals, including educators.

The survey was limited to existing CEPH-accredited graduate programs. However, given that genetics/genomics education is not a required competency for CEPH accreditation, it is plausible that programs seeking accreditation would simply not require anything excluded from what is considered the “gold standard” in public health education. It would also have been worthwhile to investigate non-accredited MPH programs to develop a clearer picture of the true status of genetics/genomics education in all MPH programs across the nation. 

The complexity of the integration of genetic/genomics into public health courses may only have been partially captured by not covering the full extent of current concerns. Other limitations include those presented through the use of an email delivery system—emails could have been lost to the spam folder, left unread, or sent to the incorrect individual. Additionally, the survey was given only in an online format, which could have precluded some responses.

### 4.4. Future Directions

Embracing the multidisciplinary approach to public health education that we advocate may help integrate genetics into public health curricula. In our study, having faculty knowledgeable in genetics/genomics was associated with related course availability. The education of current faculty members so that they can incorporate genetics/genomics into their discipline-specific courses and/or coordinate with outside disciplines is needed. To encourage this goal, continuing education courses, programs, and/or seminars could be required of current MPH program faculty members, with follow-up on what was later integrated into courses. Further, web-based courses and programs could be offered to, and possibly be required of, public health educators and the current workforce. Such courses may provide the flexibility needed to fit additional programs into current workflows. 

Future studies exploring the interest level of current MPH students in genetics/genomics would be useful in assessing the practicality of incorporation into existing programs. Confidence levels regarding the CDC’s genomics competencies can also be asked of MPH students when assessing their perceived preparedness as public health professionals and how that factor compares to programmatic responses.

## 5. Conclusions

The goal of this study was to determine the current state of genetics/genomics incorporation in CEPH-accredited MPH programs across the nation. According to this survey, the majority of programs did not offer introductory courses in public health genetics, and even if the programs had courses that contained some level of genetics/genomics, the courses were often not required for program completion. This education deficit, therefore, perpetuates an incomplete system of learning, where students starting an MPH program with little or no prior knowledge of genetics/genomics then proceed to enter the public health workforce unprepared for the current trajectory of genetics/genomics in public health.

The incorporation of public health genetics into MPH programs is imperative for the future progression of educating public health professionals. As the field of genetics/genomics grows, its applications become more relevant within all public health domains, making genetically literate public health professionals essential. 

Following analysis of this survey, we hypothesize that due to genetics/genomics not being a CEPH competency, the impetus for programs to incorporate public health genetics is simply not there. For this reason, we suggest that genetics and genomics competencies be considered during future updates of the CEPH accreditation guidelines to reflect the increasing need for genetics/genomics knowledge. Additionally, public health genetics incorporation should take a multidisciplinary approach and weave salient genetics/genomics information with existing coursework to not only emphasize its practicality but also to combat time and space barriers. The creation of continuing education programs focused on public health genetics may empower faculty members in their struggle to keep current. With the implementation of this multipronged strategy, it would be possible for practitioners to receive a more comprehensive education and, therefore, be better prepared to meet the public’s overall health needs.

## Figures and Tables

**Table 1:** Survey demographics.

Characteristic	*n* (%) ^1^
Geographical region	
Midwest	6 (24.00)
Northeast	7 (28.00)
Northwest	2 (8.00)
Southeast	5 (20.00)
Southwest	5 (20.00)
Area type	
Rural	1 (2.78)
Suburban	7 (19.44)
Urban	28 (77.78)
Years MPH program in existence (years)	
1–5	1 (2.70)
6–10	6 (16.22)
11+	30 (81.08)
Affiliated with a medical school or hospital	
Yes	20 (54.05)
No	17 (45.95)

^1^ Percentages may not add up to 100 because of missing values.

**Table 2:** Confidence levels of addressing CDC Genomics Competencies.

CDC Genomics Competency	Minimum	Maximum	Mean	Std Deviation	Variance	Count
A. Apply the basic public health sciences, (including behavioral and social sciences, biostatistics, epidemiology, informatics, and environmental health) to genomic issues, studies, and genetic testing, using the genomic vocabulary to attain the goal of disease prevention.	1	10	4.21	2.72	7.39	24
B. Identify ethical and medical limitations to genetic testing, including uses that do not benefit the individual.	1	10	4.88	2.52	6.36	25
C. Maintain up-to-date knowledge on the development of genetic advances and technologies relevant to his/her specialty or field of expertise and learn the uses of genomics as a tool for achieving public health goals related to his/her field or area of practice.	0	10	3.36	2.57	6.62	22
D. Identify the role of cultural, social, behavioral, environmental, and genetic factors in the development of disease, disease prevention, and health promoting behaviors, and their impact on medical service organization and the delivery of services to maximize wellness and prevent disease.	1	9	5.68	1.86	3.48	25
E. Participate in strategic policy planning and development related to genetic testing or genomic programs.	1	9	3.35	2.5	6.42	20
F. Collaborate with existing and emerging health agencies and organizations, academic, research, private, and commercial enterprises, including genomic-related businesses, agencies, organizations, and community partnerships to identify and solve genomic-related problems.	1	9	3.67	2.63	6.93	21
G. Participate in the evaluation of program effectiveness, accessibility, cost benefit, cost effectiveness, and quality of personal and population-based genomic services in public health.	1	10	4.14	2.57	6.6	22
H. Develop protocols to ensure informed consent and human subject protection in research.	1	10	5.83	2.96	8.75	24

**Table 3:** Statistically significant barriers to inclusion of genetics in programs.

Curriculum Characteristic		Challenged by Faculty Knowledge of Genetics/Genomics		
		Yes	No	*p*-Value
Includes one or more introductory courses in public health genetics/genomics	Yes	2	12	0.002
	No	16	7	
Includes one or more courses that are primarily focused on specific areas in public health genetics/genomics	Yes	5	14	0.009
	No	13	5	
Includes one or more courses that incorporate some topics in genetics/genomics	Yes	9	17	0.013
	No	9	2	

**Table 4:** Titles of current courses which contain an element of genetics.

Subject	Course Title	Responses
**Epidemiology**		17
	Survey of Public Health Genetics	
	Cancer Epidemiology	
	Genomic Epidemiology	
	Public Health Epidemiology	
	Infectious Disease Epidemiology	
	Chronic Disease Epidemiology	
	Measurement of Health in Epidemiology	
	Principles of Genetic Epidemiology 1&2	
	Biology of Disease in Populations	
	Foundations of Public Health	
	Public Health Genomics	
	Introduction to Genetic Epidemiology	
	Epidemiology and Natural History of Human Viral Infections	
	Genetic and Molecular Epidemiology	
	Emerging Issues in Epidemiology	
	Epidemiology	
	Research Methods in Public Health	
	Health Promotion and Disease Prevention	
	Fundamentals of Genetic Epidemiology	
	Population Based Genetics Research: Cancer Focus	
	Functional Genomics	
	Genome, Epigenome & Analysis	
**Health Policy and Ethics**		5
	Ethical Principles in Population Genetics	
	Ethical, Legal, and Social Issues in Genomics and Health	
**Health Behavior/Education**		5
	Genetics, Health Behavior and Health Education	
	Psychiatric Genomics	
	Reproductive Health	
	Introduction to Behavioral and Psychiatric Genetics	
**Biostatistics/Bioinformatics**		5
	Statistical Models and Numerical Methods in Human Genetics	
	High Throughput Molecular Genetic and Epigenetic Data Analysis	
	Statistical Population Genetics	
	Advanced Topics in Genetic Modeling	
	Statistics for Psychosocial Research: Structural Models	
**Environmental Health**		9
	Genes and the Environment	
	Environmental and Biological Science	
	Genetic Susceptibilities and Environmental Health	
	Environmental and Occupational Health	
	Environmental & Biological Fundamentals of Public Health	
	Microbiomes and Microbial Ecology in Public Health	
**Infectious Disease**		7
	Molecular Epidemiology	
	Immunology, Infection and Disease	
	Epidemiology of Diseases of Major Public Health Importance	
	Tropical Diseases	
**Nutrition**		3
	Nutrigenomics	
**Other**		10
	Introduction to Public Health Genetics	
	Genetics in Public Health	
	Seminar	
	Genetic Counseling degree offers a full range of courses	
	Biologic, Genetic and Infectious Basis of Human Disease	
	Biological Basis for Disease	

## Data Availability

The data presented in this study are available upon request from the corresponding author.

## References

[1] Center for Sharing Public Health Services Public Health. https://phnci.org/uploads/resource-files/CSII-Glossary-of-Terms-July-2019.pdf.

[2] Modell S.M., Kardia S.L.R., Citrin T., Morris M.B. (2015). Enlarging the social definition of harm to include genetics. Public Health and Harm Reduction: Principles, Perceptions and Programs.

[3] Khoury M.J., Bowen M.S., Clyne M., Dotson W.D., Gwinn M.L., Green R.F., Kolor K., Rodriguez J.L. (2018). From public health genomics to precision public health: A 20-year journey. Genet. Med..

[4] Zimmern R., Stewart A. (2006). Public health genomics: Origins and basic concepts. Ital. J. Public Health.

[5] Omenn G.S. (2000). Public health genetics: An emerging interdisciplinary field for the post-genomic era. Annu. Rev. Public Health.

[6] US Department of Health and Human Services, Office of Science Genomic Workforce Competencies 2001. https://www.cdc.gov/genomics/translation/competencies/index.htm.

[7] Institute of Medicine (2003). Who Will Keep the Public Healthy? Educating Public Health Professionals for the 21st Century.

[8] European Public Health Association Public Health Genomics. https://eupha.org/section_page.php?section_page=117.

[9] Ianuale C., Leoncini E., Mazzucco W., Marzuillo C., Villari P., Ricciardi W., Boccia S. (2014). Public Health Genomics education in post-graduate schools of hygiene and preventive medicine: A cross-sectional survey. BMC Med. Educ..

[10] Calhoun J.G., Ramiah K., Weist E.M., Shortell S.M. (2008). Development of a core competency model for the master of public health degree. Am. J. Public Health.

[11] Association of Schools of Public Health (ASPH) Education Committee Master’s Degree in Public Health Core Competency Development Project, Version 2.3. https://hpm.ph.ucla.edu/files/view/docs/ASPH_Competency_Model.pdf.

[12] American Public Health Association Our History. https://www.apha.org/about-apha/our-history.

[13] Council on Education for Public Health Schools of Public Health & Public Health Programs Accreditation Criteria. https://media.ceph.org/documents/2021.Criteria.pdf.

[14] University of Pittsburgh Graduate School of Public Health CEPH MPH Foundational Competencies. The MPH Foundational Competencies as Defined by the 2016 CEPH Accreditation Criteria. https://www.publichealth.pitt.edu/Portals/0/Main/Governance/MPH%20Committee/CEPH%20MPH%20Foundational%20Competencies.pdf.

[15] Burton H., Adams M. (2009). Professional education and training in Public Health Genomics: A working policy developed on behalf of the Public Health Genomics European Network. Public Health Genom..

[16] University of Michigan School of Public Health Certificate in Public Health Genetics. https://sph.umich.edu/genetics.

[17] University of Washington, Institute of Public Health Genetics MPH in Public Health Genetics. https://iphg.biostat.washington.edu/programs/mph.

[18] University of Pittsburgh Graduate School of Public Health MPH in Public Health Genetics. https://www.publichealth.pitt.edu/human-genetics/academics/mph.

[19] Austin M.A., Peyser P.A., Khoury M.J. (2000). The interface of genetics and public health: Research and educational challenges. Annu. Rev. Public Health..

[20] Qualtrics (2005). Qualtrics XM.

[21] StataCorp (2019). Stata Statistical Software: Release 16.

[22] Council on Education for Public Health Implementation of 2016 Criteria. https://media.ceph.org/wp_assets/implementation.pdf.

[23] Marzuillo C., De Vito C., D’Addario M., Santini P., D’Andrea E., Boccia A., Villari P. (2014). Are public health professionals prepared for public health genomics? A cross-sectional survey in Italy. BMC Health Serv. Res..

[24] Chen L.-S., Yeh Y.-L., Goodson P., Zhao S., Jung E., Muenzenberger A., Kwok O.-M., Ma P. (2019). Training Texas public health professionals and professionals-in-training in genomics. Am. J. Health Promot..

[25] Chen L.-S., Goodson P. (2009). Barriers to adopting genomics into public health education: A mixed methods study. Genet. Med..

[26] Ponte A., Greenberg S., Greendale K., Senier L. (2019). Moving the needle on action around evidence-based screening for hereditary conditions: Preparing state chronic disease directors to advance precision public health. Public Health Rep..

[27] Green R.F., Dotson W.D., Bowen S., Kolor K., Khoury M.J. (2015). Genomics in public health: Perspective from the Office of Public Health Genomics at the Centers for Disease Control and Prevention (CDC). Healthcare.

[28] Kardia S.L.R., Modell S.M., Boulton M.L., Wallace R.B. (2022). Genomic determinants of health and applications in public health and preventive medicine. Maxcy-Rosenau-Last. Public Health & Preventive Medicine.

[29] Omenn G.S. (2010). Overview of the Symposium on Public Health Significance of Genomics and Eco-genetics. Annu. Rev. Public Health.

[30] Omenn G.S., Khoury M.J., Burke W., Thomson E.J. (2000). Genetics and public health: Historical perspectives and current challenges and opportunities. Genetics and Public Health in the 21st Century.

[31] Centers for Disease Control and Prevention CDC Identifies 10 Public Health Achievements of First Decade of 21st Century. https://www.cdc.gov/media/releases/2011/p0519_publichealthachievements.html.

[32] Green E.D., Gunter C., Biesecker L.G., Di Francesco V., Easter C.L., Feingold E.A., Felsenfeld A.L., Kaufman D.J., Ostrander E.A., Pavan W.J. (2020). Strategic vision for improving human health at The Forefront of Genomics. Nature.

[33] Haga S.B., Kim E., Myers R.A., Ginsburg G.S. (2019). Primary care physicians' knowledge, attitudes, and experience with personal genetic testing. J. Pers. Med..

[34] American Public Health Association APHA Policy Statement 201012: Strengthening Genetic and Genomic Literacy. https://www.apha.org/policies-and-advocacy/public-health-policy-statements/policy-database/2014/07/30/16/37/strengthening-genetic-and-genomic-literacy.

[35] Wang G., Watts C. (2007). The role of genetics in the provision of essential public health services. Am. J. Public Health.

[36] Beskow L.M., Khoury M.J., Baker T.G., Thrasher J.F. (2001). The integration of genomics into public health research, policy and practice in the United States. Community Genet..

[37] Institute of Medicine (1988). The Future of Public Health.

[38] Vicente P., Reis E. (2010). Using questionnaire design to fight nonresponse bias in web surveys. Soc. Sci. Comput. Rev..

[39] Fowler F.J. (1988). Survey Research Methods.

[40] Chen L.-S., Goodson P. (2007). Public health genomics knowledge and attitudes: A survey of public health educators in the United States. Genet. Med..

